# HDAC1 and 2 regulate endothelial VCAM-1 expression and atherogenesis by suppressing methylation of the *GATA6* promoter

**DOI:** 10.7150/thno.55878

**Published:** 2021-03-20

**Authors:** Chengxiu Hu, Kai Peng, Qianqian Wu, Yiying Wang, Xing Fan, Dai-Min Zhang, Anthony G. Passerini, ChongXiu Sun

**Affiliations:** 1 Key Laboratory of Targeted Intervention of Cardiovascular Disease, Collaborative Innovation Center for Cardiovascular Disease Translational Medicine, Nanjing Medical University, Nanjing, China.; 2 Key laboratory of Human Functional Genomics of Jiangsu Province, Nanjing, China.; 3 Department of Cardiology, Nanjing First Hospital, Nanjing Medical University, Nanjing, China.; 4 Department of Biomedical Engineering, University of California Davis, Davis CA, USA.

**Keywords:** endothelial cell, epigenetics, atherosclerosis, vascular cell adhesion molecule-1

## Abstract

Increased expression of vascular cell adhesion molecule (VCAM)-1 on the activated arterial endothelial cell (EC) surface critically contributes to atherosclerosis which may in part be regulated by epigenetic mechanisms. This study investigated whether and how the clinically available histone deacetylases 1 and 2 (HDAC1/2) inhibitor drug Romidepsin epigenetically modulates VCAM-1 expression to suppress atherosclerosis.

**Methods:** VCAM-1 expression was analyzed in primary human aortic EC (HAEC) treated with Romidepsin or transfected with HDAC1/2-targeting siRNA. Methylation of GATA6 promoter region was examined with methylation-specific PCR assay. Enrichment of STAT3 to GATA6 promoter was detected with chromatin immunoprecipitation. Lys685Arg mutation was constructed to block STAT3 acetylation. The potential therapeutic effect of Romidepsin on atherosclerosis was evaluated in *Apoe*^-/-^ mice fed with a high-fat diet.

**Results:** Romidepsin significantly attenuated TNFα-induced VCAM-1 expression on HAEC surface and monocyte adhesion through simultaneous inhibition of HDAC1/2. This downregulation of VCAM-1 was attributable to reduced expression of transcription factor GATA6. Romidepsin enhanced STAT3 acetylation and its binding to DNA methyltransferase 1 (DNMT1), leading to hypermethylation of the *GATA6* promoter CpG-rich region at +140/+255. Blocking STAT3 acetylation at Lys685 disrupted DNMT1-STAT3 interaction, decreased *GATA6* promoter methylation, and reversed the suppressive effects of HDAC1/2 inhibition on GATA6 and VCAM-1 expression. Finally, intraperitoneal administration of Romidepsin reduced diet-induced atherosclerotic lesion development in *Apoe*^-/-^ mice, accompanied by a reduction in GATA6/VCAM-1 expression in the aorta.

**Conclusions:** HDAC1/2 contributes to VCAM-1 expression and atherosclerosis by suppressing STAT3 acetylation-dependent *GATA6* promoter methylation. These findings may provide a rationale for HDAC1/2-targeting therapy in atherosclerotic heart disease.

## Introduction

Atherosclerosis, a chronic inflammatory response of the arterial wall, underlies the pathologies of most cardiovascular diseases, which are the leading causes of morbidity and mortality worldwide. Activated endothelial cells (EC) contribute to the initiation and development of atherosclerosis largely through an increased expression of surface adhesion molecules such as vascular cell adhesion molecule (VCAM)-1 and intercellular adhesion molecule (ICAM)-1, which recruit leukocytes to the arterial wall. Compared with ICAM-1, VCAM-1 plays a major role in early atherosclerosis in mice 1 as it interacts with integrin VLA-4 on monocytes, mediating their firm adhesion onto the intima 2. Deficiency of VCAM-1 significantly reduced atherosclerotic lesions in the aorta of LDL receptor-null mice 1. Therefore, endothelial VCAM-1 expression has long been recognized as a marker of athero-inflammation and a promising therapeutic target of atherosclerotic heart diseases 2. However, little is known about its epigenetic regulation in this context although recent evidence suggests that atherosclerosis has an epigenetic component 3, 4 involving dynamic modifications of DNA and histones in both cell type- and stage-specific manners.

DNA methylation is a major epigenetic regulatory mechanism that adds a methyl group to the 5-carbon of cytosines (5-mC) usually at CpG dinucleotides, resulting in heterochromatin formation and transcriptional suppression. This process is catalyzed by DNA methyl-transferases (DNMT) and reversed by Tet methylcytosine dioxygenases. DNMT1, a member of the DNMT family, was not only required for the maintenance of normal methylation in the developing mouse embryo 5, but also contributed to aberrant CpG island methylation in cancer cells 6. Upregulation of DNMT1 by oscillatory shear or cytokine was associated with EC 7 or macrophage 8 inflammation and atherosclerosis.

Histone acetylation/deacetylation is another classical form of epigenetic modification. Similar to DNA methylation, histone deacetylase (HDAC)-catalyzed removal of the acetyl group on lysine residues within the N-terminal tails of histones is usually associated with chromatin condensation and transcriptional inhibition. More importantly, HDAC also catalyze the deacetylation of non-histone proteins located in the nucleus and the cytoplasm, which affects diverse biological functions such as enzyme activity, protein-protein interaction, DNA recruitment, and transcriptional activity 9. Mammalian HDAC are categorized into four main groups, of which class I (HDAC1/2/3 and HDAC8) and II (HDAC4/5/6/7 and HDAC9/10) constitute classical HDAC 10 and are highly expressed in EC 11. Both classes I and II were involved in the oxidative, inflammatory, and proliferative responses of EC to disturbed flow 11. However, class I HDAC exhibit much higher enzymatic activity than the class II and, in addition to histones, target diverse non-histone proteins 9 such as transcription factors 12-14 which could affect their transcriptional activities or their interaction with other proteins to alter the methylation of the gene promoter region 15.

Natural and synthetic compounds inhibiting HDAC activity were extensively studied, mainly in the treatment of cancers. To date suberoylanilide hydroxamic acid (SAHA, Vorinostat), a nonselective global HDAC inhibitor, and Romidepsin (Istodax, FK228), characterized by a relatively low IC_50_ for HDAC1 and HDAC2 (HDAC1/2), have been approved by the Food and Drug Administration (FDA) in the U.S. for treatment of hematologic cancers. HDAC isozymes including HDAC1/2 were recently reported to be upregulated in advanced human atherosclerotic tissue 16. Given the clinical availability of Romidepsin, whether this relatively specific inhibitor affects EC activation and vascular inflammation promoting atherosclerosis, and the underlying epigenetic mechanisms, warrant investigation.

In the present study, we have tested the hypothesis that Romidepsin promotes the acetylation of non-histone proteins that regulate transcription leading to a downregulation of VCAM-1 expression by EC, which is of critical functional importance to atherosclerosis. We report that Romidepsin, by inhibition of HDAC1/2, significantly suppressed TNFα-induced *GATA6* transcription and subsequent *VCAM1* expression through a mechanism involving acetylation of STAT3 and hypermethylation of the *GATA6* promoter. Moreover, administration of Romidepsin reduced diet-induced atherosclerotic lesion development in *Apoe*^-/-^ mice.

## Methods

### Animal study

All protocols were approved by the Institutional Animal Care and Use Committee (IACUC-1910021). Male *Apoe*^-/-^ mice on C57BL/6 background (The Jackson Laboratory) at 6-weeks old were randomly assigned into treatment or control group to receive i.p. injection of 2 mg/kg Romidepsin 17, 18 or the same volume of PBS once every 4 days until tissue harvest. Both groups were fed a high fat diet (Research Diet, D12108C) for 12 weeks. Mice were euthanized by CO_2_ after overnight fasting. Blood was collected to measure serum total cholesterol (Nanjing Jiancheng, A111-1), triglyceride (Nanjing Jiancheng, A110-1), high density lipoprotein cholesterol (Nanjing Jiancheng, A112-1), low density lipoprotein cholesterol and glucose (Nanjing Jiancheng, F006-1-1). To assess lesion area, heart-aorta complexes were excised and thoracic-abdominal aortas fixed with 10% formalin, while aortic sinuses and arches embedded with optimal cutting temperature for frozen section preparation 19.

### Oil red O staining

Oil red O staining was used to evaluate atherosclerotic lesion and its lipid content. For *en face* analysis, the thoracic-abdominal aorta was opened longitudinally, stained with 0.5% Oil Red O, then pinned on a silica gel with black background and photographed with a Stereomicroscope. Percentage of Oil Red O positive area was calculated using ImagePro Plus software. Cross-sections of aortic sinuses or longitudinal sections of aortic arches (5 µm) were fixed with 10% formalin, dehydrated with propylene glycol and stained with Oil Red O. Sections with 50 μm apart were mounted on the same slide. Three sections were analyzed to quantify lesion size using ImagePro Plus software. Lipid deposition was expressed as the percentage of Oil Red O positive area in the intima of sinuses and arches.

### Immunohistochemistry

Immunohistochemistry was performed as previously described 19. Briefly, serial frozen sections of mouse aortic arches were fixed in acetone. After inhibition of endogeneous peroxidase activity with 0.3% H_2_O_2_ and blocking of nonspecific binding with normal rabbit serum, tissues were incubated for 90 min with antibody against CD107b (BD Pharmingen, 553322, 1:1000) specifically for macrophages. After washing, sections were incubated with biotinylated secondary rabbit anti-rat antibody (Vector Laboratories, BA-4001, 1:200), followed by incubation with avidin-biotin-peroxidase complex (Vector Laboratories, PK-6102). The chromogen 3-amino-9-ethyl carbazole (BOSTER Biological Technology, AR1020) was used as the substrate for peroxidase. After counterstaining with hematoxylin, sections were mounted with Glycerol Gelatin (Solarbio life sciences, S2150). Images were obtained using the Inverted microscope and analyzed with the ImagePro Plus software. Macrophage contents in the intimal lesions were determined by measuring the percentage of positive areas.

### Isolation of aortic EC from mice

Primary aortic EC were isolated from mice using positive immuno-selection with a rat anti-mouse CD31, as previously described 19. Briefly, 4~8 freshly isolated aortas were minced, digested with 1 mg/mL type I collagenase (Worthington, LS004196) and filtered through 70 μm nylon filters. Anti-CD31 (BD Pharmingen, 553370)-coupled magnetic beads (Invitrogen, 11035) were used to purify EC, which were then cultured in EC growth media (EGM-2 MV Bullet Kit, Lonza, CC-3202). EC identity was evaluated by flow cytometry using FITC-conjugated rat anti-mouse CD31 (BD Pharmingen, 558738).

### Cell culture and treatment

Primary HAEC were purchased from the American Type Culture Collection (Catalog No. PCS-100-011, Lot no. 63233442, Manassas, VA) and maintained in Endothelial Growth Medium-2 (Lonza) supplemented with 10% FBS (GIBCO). HDAC inhibitor Romidepsin (HY-15149), RGFP966 (HY-13909) and PCI-34051 (HY-15224), HDAC activator ITSA-1 (HY-100508) and DNMT inhibitor Decitabine (5-Aza-2'-deoxycytidine, DCA, HY-A0004) were products of MedChemExpress. Unless otherwise indicated, HAEC up to passage 10 without serum starvation were pretreated with Romidepsin (40 nM) for 1 h and then stimulated with 0.1 ng/mL TNFα (R&D Systems) for 2 h for mRNA measurement, or for 4 h for Western blot and flow cytometry analyses.

### Cell viability assay

Cell Counting Kit-8 assay was used to examine EC viability according to the protocol recommended by the manufacturer (HY-K0301, Med Chem Express). Briefly, 10 μL WST-8 (2-(2-methoxy-4-nitrophenyl)-3-(4-nitrophenyl)-5-(2,4-disulfophenyl)-2H-tetrazolium) was added into each well of the 96-well plate where EC monolayer were grown to confluency and treated. After incubation at 37 °C for 1 h, absorbance at 450 nm and 600 nm was detected using a microplate reader ELx800 (BioTek Instruments). Absorbance at 600 nm served as a reference value.

### Cell transfection

All siRNAs were from Santa Cruz Biotechnology. The plasmid containing wild type STAT3 (pEGFP-N1-STAT3) was obtained from Addgene (#111934). To inhibit endogenous STAT3 acetylation, the coding sequence AAG for K685 was mutated to AGG (Arg, R) by site-directed mutagenesis using Site-Directed Mutagenesis Kit (Agilent) with primers: forward 5'-CAAGGAGGAGGCATTCGGAAGGTATTGTCGGCCAGAG-3' and reverse 5′-CTCTGGCCGACAATACCTTCCGAATGCCTCCTCCTTG-3′. SiRNA or plasmid was transfected with Lipofectamine 2000 (ThermoFisher Scientific). At 48-96 h posttransfection, HAEC were further treated and analyzed.

### Western blotting and immunoprecipitation analysis

For whole cell lysates, cells were washed with PBS twice and scraped on ice in 100 μL of lysis buffer supplemented with protease and phosphatase inhibitor cocktails (Roche). Immunoprecipitations were performed by using Pierce classic IP kit (26146, Thermo scientific). Whole cell lysates were collected after indicated treatments followed by pre-clear of cell lysate by incubation of the control agarose resin for 1 h at 4 °C and immunoprecipitated with Protein A/G Plus Agarose for 1 h at 4 °C. Cell lysates were then incubated with indicated antibodies overnight at 4 °C, after which the beads were washed extensively and the proteins were eluted. Samples of whole cell lysates and immunoprecipitates were boiled for 5 minutes after mixed with 1 mM dithiothreitol and 0.03% bromophenol blue. Equal amounts of total cell lysate proteins were subjected to SDS-polyacrylamide gel electrophoresis and transferred to a PVDF membrane (Millipore) before blocked by 5% fat-free milk or 1% BSA. Membranes were then incubated with primary antibodies overnight at 4 °C and with horseradish peroxidase-conjugated secondary antibodies for 2 h at room temperature. Target bands were visualized with ECL (Tanon) and a digital gel image-analysis system. Primary antibody targeting VCAM-1 (ab134047) was purchased from Abcam and anti-ICAM-1 antibody (sc-8439) from Santa Cruz Biotechnology. Antibodies targeting HDAC1 (34589), HDAC2 (57156), GATA6 (5851), IRF-1 (8478), phospho-Ser^536^ NF-κB (3036), NF-κB (8242), STAT3 (12640), phospho-Tyr^705^ STAT3 (9145), acetyl-Lys^685^ STAT3 (2523), acetyl-Lys^27^ Histone H3 (#4353), and GAPDH (2118) were purchased from Cell Signaling Technology. Histone H3 antibody (BS1751) was from Bioworld Technology. Band densities were quantified using ImageJ software (National Institutes of Health) by normalization to GAPDH.

### Quantification of mRNA and heterogeneous nuclear RNA (hnRNA)

After treatment, total RNA in HAEC was extracted using TRIzol reagent and was reverse transcribed into cDNA using the PrimeScript RT kit (Takara Biotechnology), followed by real-time PCR using SYBR Green (Roche) on a LightCycler 480 Instrument II (Roche). Relative gene expression was normalized to the GAPDH mRNA level with the 2^-ΔΔCt^ method where Ct is threshold cycle. The primers for mRNA measurement were as follows: *HDAC1*, forward 5'- CTACTACGACGGGGATGTTGG-3' and reverse 5'-GAGTCATGCGGATTCGGTGAG-3'; *HDAC2*, forward 5'-ATGGCGTACAGTCAAGGAGG-3' and reverse 5'-TGCGGATTCTATGAGGCTTCA-3'; *GATA6*, forward 5'-TCAAACCAGGAAACGAAAACC-3' and reverse 5'-TCAAACCAGGAAACGAAAACC-3'; hn*GATA6*, forward 5'-CCAGGATTGTAACCGCTTCTC-3' and reverse 5'-CTTGACCCGAATACTTGAGC-3'; *VCAM1*, forward 5'-AACCTTGCAGCTTACAGTGA-3' and reverse 5'-TGTGTGAAGGAGTTAATTTGATTGG-3'; *ICAM1*, forward 5'-CGCTGAGCTCCTCTGCTACT-3' and reverse 5'-TAGGCAACGGGGTCTCTATG-3'; *IRF1*, forward 5'-GTCCAGCCGAGATGCTAAGAG-3' and reverse 5'-TGGTCATCAGGCAGAGTGGAG-3' and *GAPDH*, forward 5'- GAGTCAACGGATTTGGTCGT-3' and reverse 5'-GGTGCCATGGAATTTCCAT-3', respectively.

### Luciferase activity assay

*VCAM1* -288/+12 was amplified by PCR using forward and reverse primers containing restriction sites for MluI and XhoI and inserted into pGL3 firefly luciferase reporter vector (Promega). The Site-Directed Mutagenesis Kit (Agilent) was used to generate *VCAM1* -288/+12 GATA mut (with the GATA -259 site mutated). Luciferase reporter plasmids of NF-κB (11501ES03) and GATA (11525ES03) were obtained from Yeasen Biotech (Shanghai, China). HAEC growing on a 24-well plate were transfected with 0.8 μg of each construct using Lipofectamine 2000 together with 0.1 μg phRL-TK (Promega) containing Renilla luciferase-coding sequence, which served as an internal transfection control. Luciferase activity was measured with Dual-Luciferase Reporter system (Promega) on a GloMax 20/20 Luminometer (Promega).

### Chromatin immunoprecipitation assay

HAEC or mouse aortic EC at 70-80% confluence were cultured in 15 cm plates and treated with Romidepsin (40 nM) for 1 h followed by stimulation with (1 ng/mL) human TNFα or 10 ng/mL mouse TNFα for 4 h. The preparation and immunoprecipitation of chromatin was processed by using ChIP-IT Express kit (Active Motif, 53008). GATA6 promoter binding was quantified by quantitative real-time PCR and normalized to input DNA. As predicted by the JASPAR, binding of human STAT3 to two potential sites on GATA6 promoter (-762 CT CTCCAGGGAAA -752 and +84 TTTTCCGGCAG +94) was examined. The primers were: forward 5'-TTAGGGCTCGGTGAGTCCAATC-3' and reverse 5'-TCTTACTGCTCTGCCGGAAAACT-3'; forward 5'-TGATAACTGTTTGGAGGGAGC-3' and reverse 5'-ACCTTTGGGAACTTTAACTCG-3'. Enrichment of human DNMT1 to the hypermethylated +140/+255 region of GATA6 promoter was examined using the primers: forward 5'- CTTTCCTCCCCTCCACCCCTACTC-3' and reverse 5'- GAAGTTGGTCCGCGGTGTCCC-3'. Mouse STAT3 binding to* Gata6* promoter (-1479 TTCAAGGAAA -1470) and GATA6 binding to *Vcam1* promoter (-271 TATAAAAATAAGAACTA -255) were examined using the primers: forward 5'- TTCTCCCGCAGCACA-3' and reverse 5'- TTCCTGGAAGCATTTGA-3'; forward 5'- CTGCATCAACGTCCT-3' and reverse 5'- GACAGCAAAGACAGAG-3', respectively.

### Flow cytometry

HAEC were detached using an enzyme-free cell dissociation buffer (GIBCO) and labeled with FITC-conjugated anti-human VCAM-1 antibody or PerCP-Cy5.5-conjugated anti-human ICAM-1 antibody (BD Pharmingen, BD Biosciences). Cells were then analyzed by a FACSCalibur flow cytometer (BD Biosciences). FlowJo software was applied to post-acquisition analysis.

### Immunofluorescent staining

HAEC were grown on coverslips to confluence. After pretreatment with Romidepsin, HAEC were stimulated with TNFα for 4 h before fixation, permeabilization, and incubation with rabbit anti-human STAT3 antibody (12640, Cell Signaling Technology). After staining with Alexa Fluor 488-conjugated donkey anti-rabbit IgG (711-545-152, Jackson ImmunoResearch) cells were counterstained with DAPI. Images were captured by a confocal laser scanning microscope (FV1200), and FITC intensity of cells was quantified with Image-Pro Plus 6.0 (Media Cybernetics).

### Monocyte adhesion

Fluorescent dye DiO-labeled THP-1 cells (5×10^4^, American Type Culture Collection) were cocultured with TNFα-stimulated HAEC in a 12-well plate for 10 min. After three washes with PBS, THP-1 cells adhered onto the HAEC monolayer were identified by positive DiO fluorescence under a fluorescence microscope.

### DNA methylation assay

The methylation status of *GATA6* promoter was assessed using methylation-specific PCR (MSP) assay. Bisulfite modification of 100 ng DNA preceding MSP was performed using the EpiTect Bisulfite Kit (Qiagen, 59104) following the manufacturer's protocol. CpG Methyltransferase M. SssI (New England Biolabs, M0226S)-treated peripheral blood leukocyte DNA was used as a reference sample for complete methylation. PCR primers targeting the methylated (M) or unmethylated (U) cytosines in the five CpG-rich regions were designed with Methprimer. *GATA6* promoter methylation was evaluated by agarose gel electrophoresis detection of PCR products. The quantification was performed with real-time PCR with methylated cytosines-targeting primers followed by normalization to* UBB* expression.

MSP1-M, forward 5'-GTTATTTTTTTTGGGAGTCGC-3' and reverse 5'-ATTTCAACGTAACCGCATTT-3'; MSP1-U, forward 5'-TTGGTTATTTTTTTTGGGAGTTGT-3' and reverse 5'- CCAATTTCAACATAACCACATTT-3'; MSP2-M, forward 5'- TTTTTGGGGTTACGTTTGTC-3' and reverse 5'-TATCAACGCCGATCTATCAA-3'; MSP2-U, forward 5'-AGTTTTTTGGGGTTATGTTTGTT-3' and reverse 5'-TATCAACACCAATCTATCAACAA-3'; MSP3-M, forward 5'-TTAGGGATATTAAAAGTTGGAGAGC-3' and reverse 5'-TCGAAATACTACGACTCAAATCGTA-3'; MSP3-U, forward 5'-TTAGGGATATTAAAAGTTGGAGAGTGT-3' and reverse 5'-TCAAAATACTACAACTCAAATCATA-3'; MSP4-M, forward 5'-GGGTTTGCGGTTTAGTTTAC-3' and reverse 5'-AACTACGCTCAACGAACAAC -3'; MSP4-U, forward 5'-ATAGGGTTTGTGGTTTAGTTTAT-3' and reverse 5'- AAAACTACACTCAACAAACAACT-3'; MSP5-M, forward 5'-CGGCGTAGATTTCGGATTCGC-3' and reverse 5'-CAACCGAACCTCGAACGAACG-3'; MSP5-U, forward 5'-GTGTGGGGTAGATTTTGGATTTGT-3' and reverse 5'-AAACAACCAAACCTCAAACAAACA-3'; UBB, forward 5'-ATAGTGGGTTTTGTTGATTTGA-3' and reverse 5'-CCTTTCTCACACTAAAATTCCA-3'.

### Statistical analysis

Data are presented as means ± SE (GraphPad Prism). For comparison between two groups, t-test was used. To analyze multiple groups, ANOVA with a Dunnett's or Tukey's posttest was applied. P≤0.05 was considered significant.

## Results

### HDAC1/2 activity was required for TNFα-induced VCAM-1 expression and monocytic adhesion on HAEC

**Figure 1:**
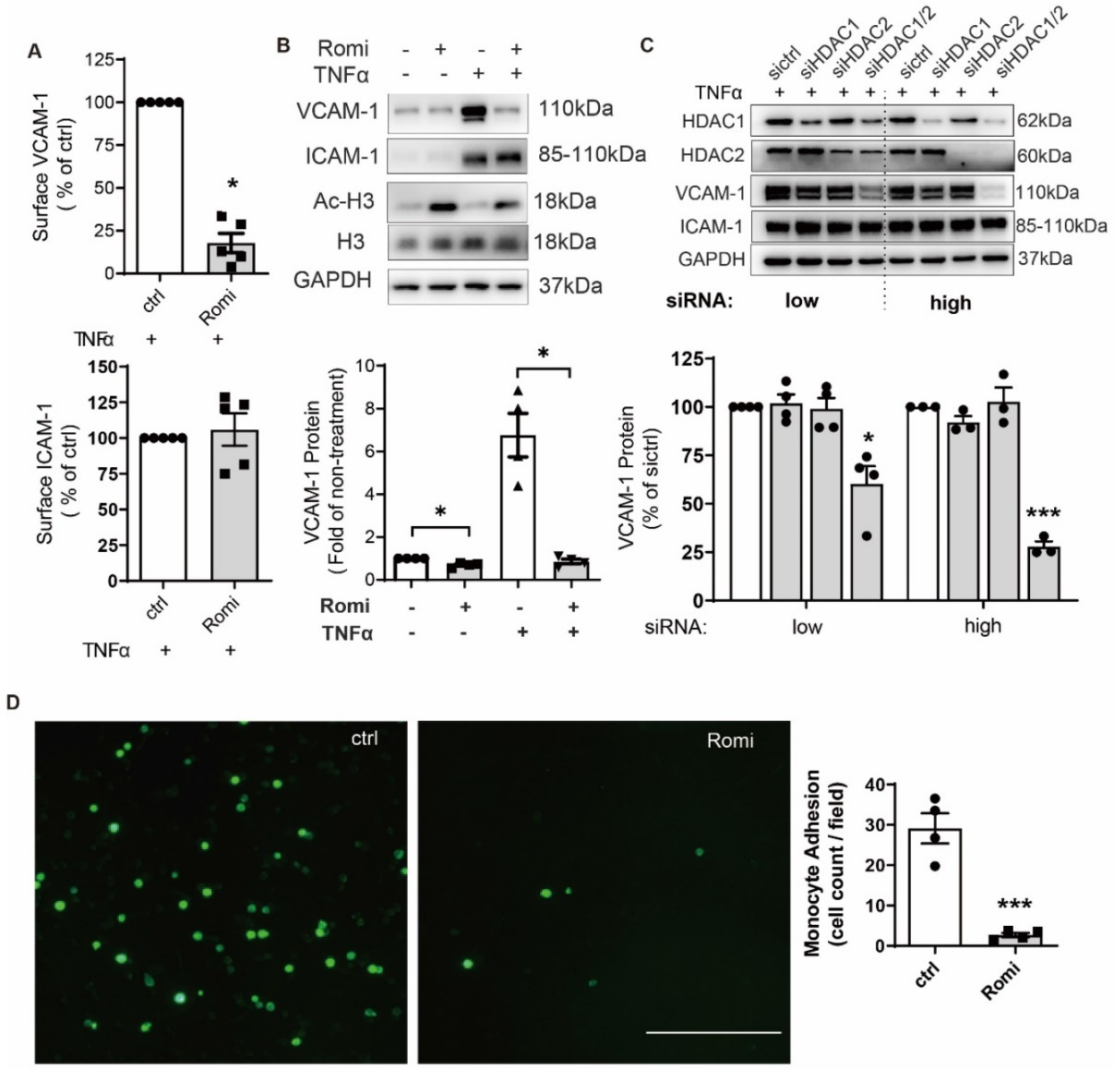
** Inhibition of HDAC1/2 attenuated TNFα-induced VCAM-1 surface expression on HAEC and subsequent monocyte adhesion.** (**A-B**) HAEC were treated with 40 nM Romidepsin (Romi) for 1 h before incubation with 0.1 ng/mL TNFα for 4 h followed by flow cytometry (n = 3, A) and Western blot analysis (n = 4, B) of VCAM-1 and ICAM-1 expression. Increase in the acetylation level of HDAC substrate Histone H3 (Ac-H3) confirms the activity of Romidepsin. Shown below is quantification of VCAM-1 protein (**C**) HAEC were transfected with 20 nM (low) or 50 nM (high) control siRNA (sictrl), or siRNA targeting HDAC1 (siHDAC1), HDAC2 (siHDAC2) or both (siHDAC1/2) before stimulation with 0.1 ng/mL TNFα for 4 h followed by Western blot analysis. VCAM-1 expression was quantified with Image J (n = 4). (**D**) HAEC were pretreated with Romi followed by stimulation with TNFα as described above. Adhered DiO-stained THP-1 cells were visualized by fluorescence microscopy and quantified (n = 4). Scale = 100 μm. *p < 0.05; ***p < 0.001, one sample *t*-test (A, B); paired two-tailed *t*-test (D); repeated measures one-way ANOVA followed by Dunnett's test (vs. sictrl, C).

Romidepsin inhibits HDAC1/2 with an IC_50_ of 36 nM and 47 nM, respectively 20. Therefore, a dose of 40 nM was used in this study to ensure the specific inhibition on HDAC1/2. CCK-8 assay indicated that 1 h pretreatment at this dose followed by another 2 h or 4 h co-treatment with TNFα did not affect HAEC viability (Figure S1A). Flow cytometry (Figure 1A) indicated that 1 h pretreatment with 40 nM Romidepsin inhibited 0.1 ng/mL 21 TNFα-induced VCAM-1 surface expression by ~82%, consistent with a dramatic decrease in the total protein revealed by Western blotting (Figure 1B).

In contrast to VCAM-1, ICAM-1 expression was not affected. In line with effect of pharmacological inhibition, siRNA-mediated dual depletion of HDAC1/2 decreased VCAM-1 expression. The magnitude of the inhibitory effect was dependent on the amount of siRNA (Figure 1C). However, separate depletion of *HDAC1* or *HDAC2* produced no such effect (Figure 1C). In contrast to inhibition of HDAC1/2, treatment with HDAC activator ITSA-1 increased VCAM-1 expression (Figure S1B).

Romidepsin was characterized as a class I HDAC inhibitor. In addition to HDAC1/2, Romidepsin also inhibits class I members HDAC3 and HDAC8, although to a lesser extent 22. However, the specific inhibition of HDAC3 or HDAC8 with its respective inhibitor RGFP966 or PCI-34051 at a dose above the IC_50_ did not cause significant inhibition of VCAM-1 expression (Figure S1C), confirming the HDAC1/2-specific effect of Romidepsin.

Consistent with the recognized role of VCAM-1 in mediating monocyte adhesion 2, Romidepsin significantly suppressed adhesion of monocytic THP-1 cells onto TNFα-stimulated HAEC monolayers (Figure 1D). These results suggest the specificity of HDAC1/2 in regulating a pathway leading to cytokine-induced VCAM-1 expression and the subsequent monocytic adhesion onto HAEC.

### HDAC1/2 regulated *VCAM1* transcription through a mechanism involving transcription factor GATA6

**Figure 2:**
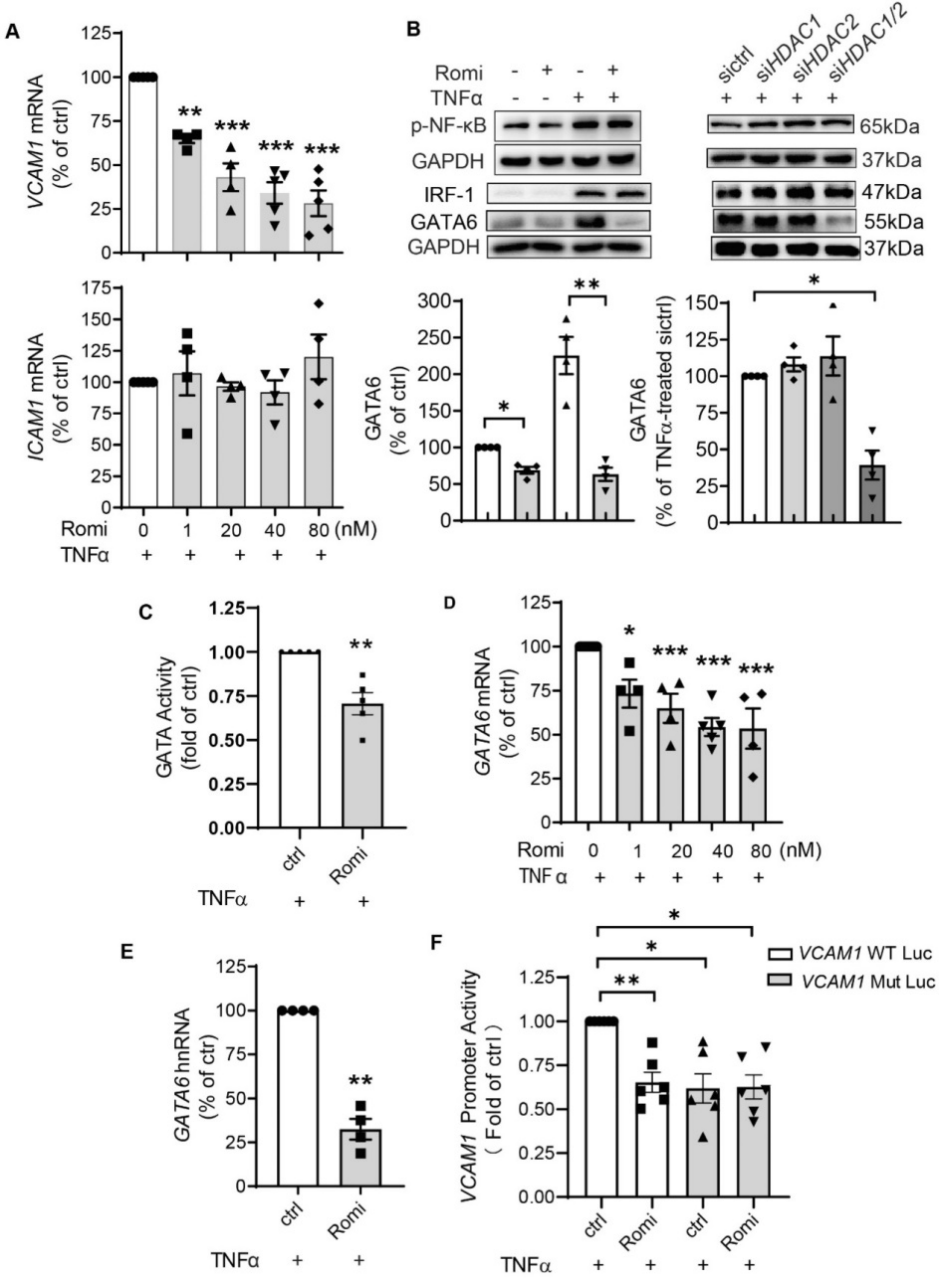
**HDAC1/2 regulated *VCAM1* transcription through a mechanism involving transcription factor GATA6.** (**A**) HAEC were pretreated with Romi (40 nM) at indicated doses for 1 h and co-cultured with 0.1 ng/mL TNFα for 2 h prior to quantitative PCR to measure *VCAM1* and *ICAM1* mRNA (n = 4-5). (**B**) After treatment with Romi or transfection with siRNA, HAEC were stimulated with TNFα for 4 h prior to Western blot analysis (n = 3). Shown below is quantification of GATA6 protein. (**C**) HAEC were transfected with luciferase reporter plasmid of GATA, treated as in (A) followed by dual-luciferase assay to detect the transcriptional activity of GATA (n = 4). (D) HAEC were treated as in (A), then steady-state *GATA6* mRNA was quantified (n = 4-5). (**E**) After sequential treatment with Romi (40 nM) and stimulation with TNFα (0.1 ng/mL), heterogeneous nuclear *GATA6* RNA (*GATA6* hnRNA) (n = 4) was analyzed. (**F**) Activity of pGL3 firefly luciferase (luc) construct containing wild type *VCAM1* promoter or GATA-binding site mutation (*VCAM1* Mut) was measured with dual-luciferase reporter system (n = 5). *p < 0.05; **p < 0.01; ***p < 0.001 vs ctrl repeated measures one-way ANOVA followed by Dunnett's test (A, D) or by Tukey's test (B, F); one sample *t*-test (C, E).

Quantitative PCR indicated that Romidepsin blocked both basal (Figure S1D-E) and TNFα-induced *VCAM1* but not *ICAM1* transcription in a dose-dependent manner (Figure 2A), with a 66% inhibition occurring at 40 nM. As the IC_50_s for Romidepsin to inhibit class II HDAC isoenzymes such as HDAC4 and HDAC6 are >500 nM 22, this together with the results of HDAC3/8 inhibition (Figure S1C), further indicated that the observations associated with the dose (40 nM) applied in this study were attributable to HDAC1/2 inhibition.

To investigate how HDAC1/2 transcriptionally regulate *VCAM1*, the activity of *VCAM1*-binding transcription factors was examined. It is known that both *VCAM1* and *ICAM1* promoters contain binding sites for transcription factors such as NF-κB and AP-1. However, binding sites for transcription factors IRF-1 and GATA6 only reside in *VCAM1* promoter and account for differential regulation of VCAM-1 and ICAM-1 expression 23-26. Consistent with the observation that HDAC1/2 inhibition did not change ICAM-1 expression, Western blotting and dual-luciferase assay indicated that both NF-κB p65 phosphorylation (Figure 2B) and its transcriptional activity (Figure S1F) were not affected.

However, inhibition of HDAC1/2 downregulated basal and especially TNFα-stimulated GATA6 protein expression (Figure 2B), which was not seen with HDAC3 or HDAC8 inhibition (Figure S1C). In line with the change at the protein level, the transcriptional activity of GATA was decreased (Figure 2C). The basal GATA6 transcription was downregulated by Romidepsin treatment (Figure S1G). Moreover, similar to the effect on *VCAM1* mRNA (Figure 2A), Romidepsin inhibited TNFα-induced *GATA6* transcription in a dose-dependent manner with an inhibition by 50% at the dose of ~40 nM (Figure 2D). In addition to the state-steady *GATA6* mRNA, heterogeneous nuclear RNA (hnRNA) expression was suppressed (Figure 2E), confirming the suppression of *GATA6* transcription. In contrast to GATA6, neither IRF-1 protein (Figure 2B) nor mRNA expression (Figure S1H) was significantly affected by inhibition of HDAC1/2.

Of the two consensus GATA binding sites on the human* VCAM1* promoter region at ^_^244 and^ _^259 upstream of the start site of transcription (TSS) 27, the -259 site is the main sequence used by EC for binding GATA6 in response to TNFα stimulation 28. Accordingly, *VCAM1* promoter activity was examined with luciferase construct containing wild type sequence or sequence with the -259 site mutated (Figure S1I). Consistent with previous results by others 28 and ourselves 21, the mutation of -259 site trended to reduce the basal activity (Figure S1J). Moreover, this mutation almost halved TNFα-amplified VCAM1 promoter activity (Figure 2F). Romidepsin treatment inhibited the activity of wild type but not that of mutant either in the absence or presence of TNFα.

Together these data clearly demonstrate that inhibition of HDAC1/2 significantly attenuated TNFα-induced GATA6 transactivation, thereby inhibiting GATA6-mediated VCAM-1 expression in HAEC.

### HDAC1/2 modulated the methylation of *GATA6* promoter region

**Figure 3:**
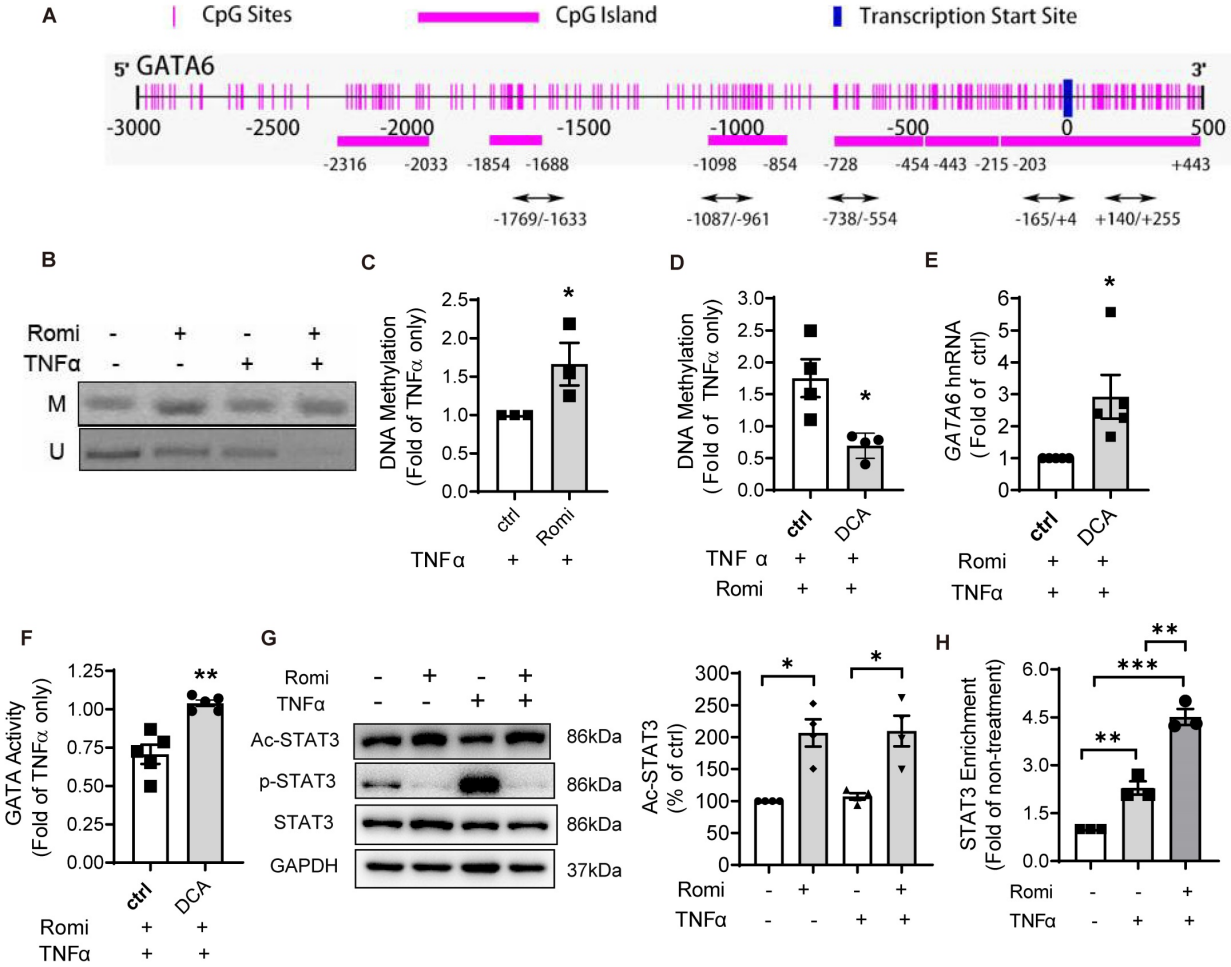
** HDAC1/2 modulated the methylation of *GATA6* promoter region and enrichment of STAT3 to *GATA6* promoter.** (**A**) Schematic illustration of seven CpG islands in human *GATA6* promoter. There are two CpG islands contained in -203/+443. Primers were designed to amplify the five CpG-rich regions. (**B**) After treatment, *GATA6* promoter methylation in CpG-rich region +144/+255 was evaluated by methylation-specific PCR (MSP) assay with primers targeting the methylated (M) or unmethylated (U) cytosines. Shown are representative agarose gel electrophoresis images. (**C**) *GATA6* methylation at +144/+255 was quantified with real-time PCR with methylated cytosines-targeting primers and normalized to *UBB* expression (n = 3). (**D-F**) HAEC were pretreated with Romidepsin alone or together with DCA (5 µM) for 1 h followed by stimulation with TNFα. (**D**) *GATA6* promoter methylation in +144/+255 region was quantified as in (C) (n = 4). (**E**) *GATA6* hnRNA expression was measured as in 2E (n = 5). (**F**), GATA activity was examined as in 2F (n = 5). (**G**) HAEC were pretreated with Romi for 1 h before stimulation with TNFα for 4 h. The phosphorylation at Tyr705 and acetylation at Lys685 of STAT3 were then analyzed with Western blotting (n = 4). Shown right is quantification of Ac-STAT3 protein. (**H**) HAEC were incubated with Romi for 1 h followed by 4 h treatment with 1 ng/mL TNFα. A chromatin immunoprecipitation assay was applied to examine enrichment of STAT3 onto +84/+94 site of *GATA6* promoter (n = 3). *p < 0.05; **p < 0.01; ***p < 0.001 vs ctrl, two-tailed *t*-test, C, D, F); one sample* t*-test (E); repeated measures one-way ANOVA followed by Tukey's test (G, H).

Since promoter methylation is an important epigenetic gene-silencing mechanism 29, the methylation status of* GATA6* promoter was examined. The Methprimer program predicted seven CpG island located at the promoter region of human* GATA6* (Figure 3A), spanning from 2000bp upstream to 500bp downstream of TSS. MSP assay was conducted using specific primers targeting both the methylated and unmethylated cytosines in these CpG sites. In total, 10 pairs of primers targeting five CpG-rich regions including -1769/-1633, -1087/-961, -738/-554, -165/+4 and +140/+255, were designed. As shown in Figure 3B-C, Romidepsin treatment significantly enhanced *GATA6* promoter methylation but decreased the unmethylated level of +140/+255 especially with TNFα treatment. Quantitative PCR revealed a 66% increase in the methylation of this region (Figure 3C). However, methylation was not detectable in any of other four regions (Figure S2A). To further confirm the negative regulation of methylation on* GATA6* promoter activity, the DNA methyltransferase inhibitor Decitabine (5-Aza-2'-deoxycytidine, DCA) was applied to inhibit DNA methylation. MSP assay confirmed Romidepsin-caused hypermethylation of *GATA6* +140/+255 was attenuated in DCA-treated HAEC (Figure 3D). As expected, this treatment rescued GATA6 transcription suppressed by Romidepsin either in the absence (Figure S2B) or presence of TNFα (Figure 3E). Consistently with the increased expression, inhibition of DNA methylation increased the transcriptional activity of GATA (Figure S2C), or rescued its activity suppressed by Romidepsin (Figure 3F).

### HDAC1/2 enhanced STAT3 acetylation and its binding to *GATA6* promoter region

Previous studies have demonstrated that HDAC and their inhibitors regulate the acetylation of well-known non-histone proteins that act as transcription factors, including STAT3 12, p53 13 and NF-κB 14, thus modulating gene expression. Moreover, acetylated STAT3 was revealed to cause gene silencing through enhancing the methylation of estrogen receptor-α gene promoter in cancer cells 15.

Bioinformatics analysis suggested potential binding sites for two transcription factors, STAT3 and AP-2α, in the regions proximal to +140/+255 of *GATA6* promoter. Western blot analysis with acetyl-STAT3-specific antibody indicated that the acetylation at Lys685 was elevated by Romidepsin (Figure 3G) while the total protein level was not affected; however, acetylation of AP-2α was not detected in the anti-AP-2α immuno-precipitate using an acetylated-Lysine antibody (data not shown).

STAT3 is a recognized transcriptional factor for *GATA6*. In addition to +84/+94bp which was proximal to the methylation region +140/+255, another binding site at -762/-752 relative to TSS was also predicted (Figure S3A). ChIP assay confirmed the interaction of STAT3 at the promoter region containing +84/+94 but not at that corresponding to -762/-752. The enrichment of STAT3 to this region was increased 1.3-fold by TNFα stimulation (Figure 3H). Furthermore, the suppressive effect of Romidepsin on *GATA6* transcription was not due to reduction in the interaction of STAT3 to *GATA6* promoter. On the contrary, Romidepsin doubled its enrichment, concomitant with an increase in STAT3 localization to the nuclei (Figure S3B-C).

These findings suggest that inhibition of HDAC1/2 increase the acetylation of STAT3, which, by binding to *GATA6* promoter, may cause hypermethylation of the CpG-rich region to suppress GATA6 expression.

### STAT3 acetylation contributed to the suppressive effect of HDAC1/2 inhibition on GATA6 and VCAM-1 independent of phosphorylation

**Figure 4:**
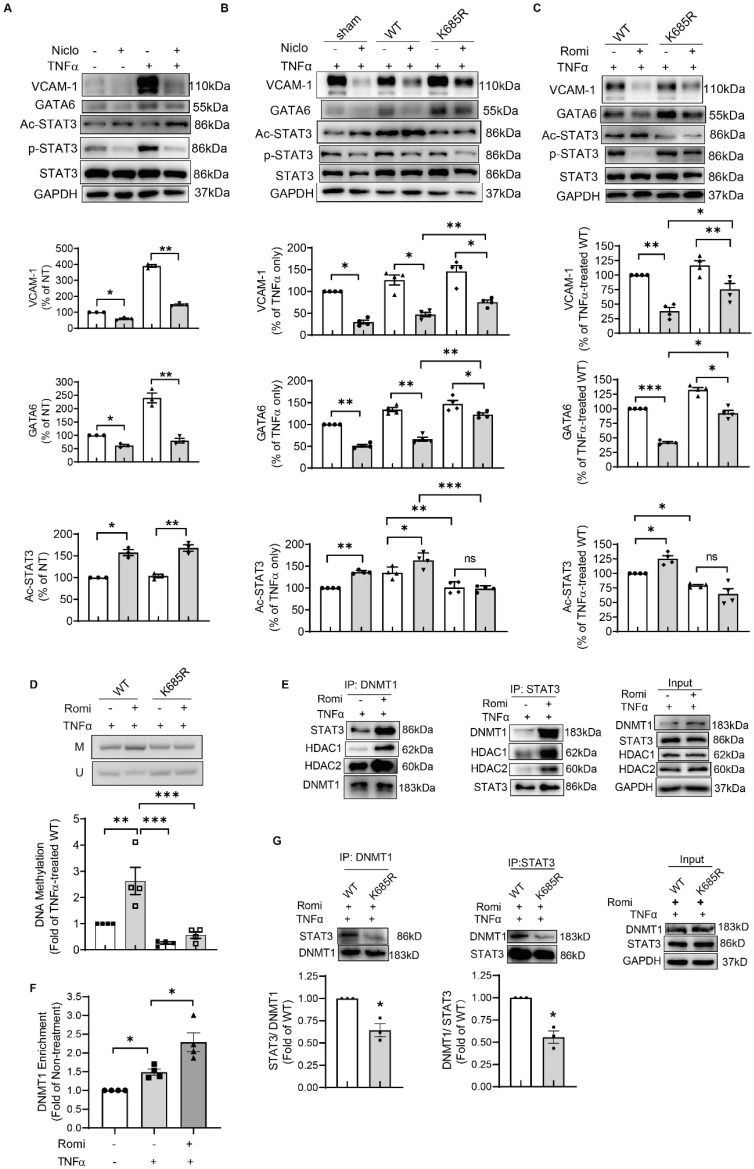
** Blocking STAT3 acetylation at Lys685 disrupted DNMT1-STAT3 interaction and reversed the suppressive effects of HDAC1/2 inhibition on GATA6 and VCAM-1 expression in HAEC. (A)** HAEC were pretreated with Niclosamide (2 μM, Niclo) for 1 h, stimulated with TNFα for 4 h followed by Western blotting (n = 4). (**B-C**) After transfection with empty vector (sham), vector containing wild type STAT3-coding sequence (WT), or sequence bearing K685R mutation (K685R), HAEC were pretreated with Niclo (C) or Romi (D) for 1 h and stimulated with TNFα for 4 h prior to Western blot analysis (n = 3). For A-C, shown below is quantification of VCAM-1, GATA6 and Ac-STAT3 protein. (**D**) After overexpression with WT STAT3 or K685R mutant, HAEC were treated as above followed by MSP assay to evaluate *GATA6* methylation at +144/+255 with agarose gel electrophoresis and real-time PCR (n = 4) that was carried out with methylated cytosines-targeting primers and the data were normalized to *UBB* expression. (**E**) HAEC were pretreated with Romi and stimulated with TNFα, followed by immunoprecipitation with anti-DNMT1 or anti-STAT3 antibody (n = 3). (**F**) After treatment, enrichment of DNMT1 onto +140/+255 of *GATA6* promoter was evaluated with chromatin immunoprecipitation assay (n = 3). (**G**) HAEC were transfected with WT STAT3 or K685R mutant, pretreated with Romi and stimulated with TNFα, followed by immunoprecipitation as above (n = 3). Shown are representative images. The ratio between STAT3 and DNMT1 was quantified and shown below. *p < 0.05, **p < 0.01, **p < 0.001, repeated measures one-way ANOVA followed by Tukey's test (A, B, C, D, F); one sample t-test (G).

Treatment with Niclosamide to specifically inhibit STAT3 activity suppressed TNFα induction of VCAM-1 and GATA6 expression (Figure 4A), confirming the role for STAT3 in the transcriptional regulation of *GATA6*. Since the discovery of STAT3, studies have demonstrated the importance of phosphorylation in regulating its transcriptional function. Interestingly, Niclosamide known to suppress STAT3 transcriptional activity through impacting its phosphorylation at Tyr705 30, also enhanced STAT3 acetylation at Lys685 in HAEC (Figure 4A). Similarly, Romidepsin increased acetylation of STAT3, concomitant with decreased STAT3 phosphorylation (Figure 3G).

To solidify the contribution of STAT3 acetylation to the effects conferred by inhibition of HDAC1/2, an acetylation-deficient STAT3 mutant in which Lys685 was replaced by an Arg (K685R) was expressed in HAEC. Western blot analysis revealed that this mutation partially reversed GATA6 and VCAM-1 protein production inhibited by Niclosamide or Romidepsin (Figure 4B and 4C). Consistently, *GATA6* promoter methylation at +140/+255 region was decreased by K685R mutation (Figure 4D).

Romidepsin treatment failed to inhibit STAT3 phosphorylation in K685R mutant-overexpressed HAEC (Figure 4C), suggesting that the low phosphorylation state of STAT3 in Romidepsin-treated HAEC might be due to high acetylation. However, K685R mutation was still able to reverse the suppressive effect of Niclosamide on GATA6 and VCAM-1 expression (Figure 4B) although blocking STAT3 acetylation by the mutation had little effect on the capacity for Niclosamide to inhibit STAT3 phosphorylation. Together these results indicate that STAT3 acetylation contributed to the inhibitory effect of HDAC1/2 inactivation on GATA6 and VCAM-1 independent of phosphorylation.

### The acetyl STAT3 colocalized with DNMT1 to enhance methylation of *GATA6*

To further investigate how acetylation of STAT3 modulated *GATA6* promoter methylation, we assessed whether HDAC1/2 affected STAT3 interaction with DNMT1 that binds to chromatin and bears the main responsibility for the maintenance of DNA methylation. Western blotting following immunoprecipitation with anti-STAT3 antibody indicated that Romidepsin indeed promoted the association between STAT3 and DNMT1 (Figure 4E). ChIP assay confirmed the recruitment of DNMT1 to +140/+255 region of *GATA6*, which was increased by Romidepsin (Figure 4F). The STAT3- DNMT1 interaction was weakened by K685R mutation (Figure 4G). Moreover, both HDAC1 and HDAC2 were present in the immuno-precipitate (Figure 4E), confirming the involvement of a HDAC1/2 complex in the regulation of STAT3 acetylation and gene expression. Interestingly, Romidepsin treatment enhanced HDAC1/2 association with STAT3-DNMT1 complex. Taken together, these results suggest that inhibition of HDAC1/2 lead to acetylation of STAT3 which recruited DNMT1 to *GATA6* promoter region in an acetylation-dependent manner to suppress GATA6 and VCAM-1 expression.

### Inhibition of HDAC1/2 reduced diet-induced atherosclerotic lesion development in *Apoe*^-/-^ mice

**Figure 5:**
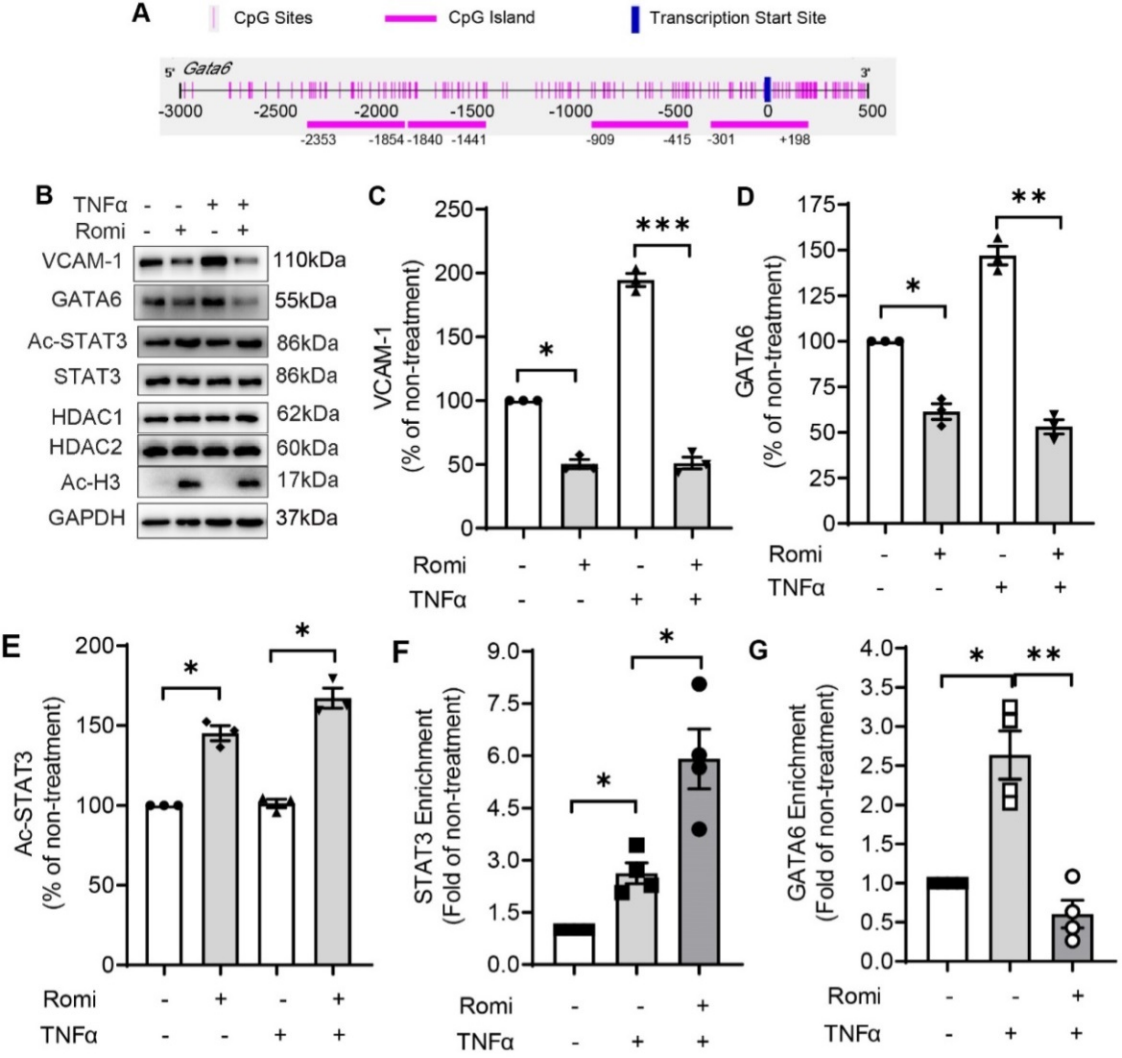
** Inhibition of HDAC1/2 attenuated TNFα-induced VCAM-1 expression in primary EC isolated from mouse aorta.** (**A**) Schematic illustration of CpG islands in mouse *Gata* promoter. (**B**) EC were isolated from aorta of *Apoe*^-/-^ mice. After 80-90% confluency, they were pretreated with Romi, and stimulated with murine TNFα (10 ng/mL) followed by Western blot analysis (n = 3). Increased Ac-H3 level confirmed Romi activity. (**C-E**) VCAM-1 (C), GATA6 (D) and Ac-STAT3 was quantified. (F-G) Mouse primary EC were incubated with Romi for 1 h followed by 4 h treatment with 10 ng/mL TNFα. A chromatin immunoprecipitation assay was applied to examine enrichment of STAT3 onto -1479/-1470 site of *Gata6* promoter (F) and GATA6 onto -271/-255 site of *Vcam1* promoter (G) (n = 4).

Similar to human* VCAM1* gene, murine* Vcam1* promoter region contains several potential GATA6 binding motifs as predicted by JASPAR program. The one with the highest score is located at -271/-255. Moreover, the Methprimer program predicted several CpG island located at the promoter region of murine* Gata6* (Figure 5A). One of them (-1840/-1441) was further predicted by JASPAR to contain STAT3-binding site (-1479/-1470). Moreover, mouse STAT3 is 100% homologous to its human counterpart as predicted by BLASTP program. These *in silico* analysis results suggested both human and mouse EC might share the mechanism in regulating VCAM-1 expression.

Supporting this notion, Romidepsin treatment significantly inhibited basal or 10 ng/mL murine TNFα-induced VCAM-1 and GATA6 expression concomitant with an elevation in the acetylation of STAT3 in primary EC isolated from mouse aortae (Figure 5B-E). Furthermore, in accordance with the observation in HAEC, Romidepsin treatment increased STAT3 binding to CpG rich -1840/-1441 region of *Gata6* promoter (Figure 5F) accompanied by a decrease in GATA6 binding to *Vcam1* promoter (Figure 5G).

**Figure 6:**
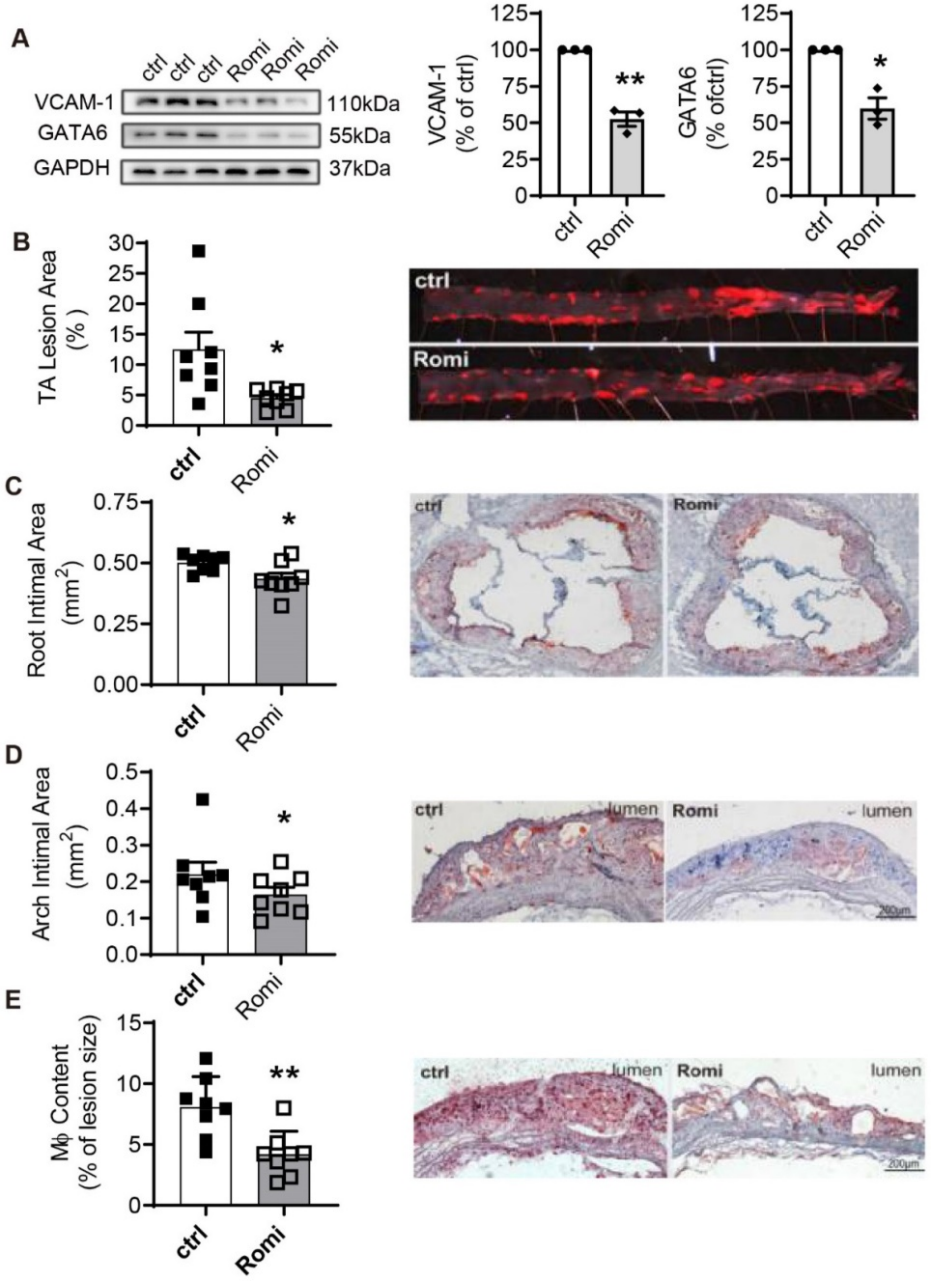
** Inhibition of HDAC1/2 alleviated diet-induced atherosclerotic lesion development in *Apoe*^-/-^ mice.** *Apoe*^-/-^ male mice were i.p. injected with Romi (2 mg/kg body weight) or PBS (ctrl) and fed with a high-fat diet for 12 weeks followed by VCAM-1 and GATA6 expression in the thoracic abdominal artery analyzed with Western Blotting and (**B-E**) atherosclerosis characterized. (**B**) *En face* staining of lesion areas with Oil Red O in thoracic-abdominal aorta (TA) (n = 8). (**C**) Cross-sections of aortic sinuses were stained with Oil Red O for intimal area and lipid deposition (n = 8). (**D-E**) Longitudinal sections of aortic arches were stained with Oil Red O (**D**) or immunostained for macrophages (Mφ) with anti-Mac-3 antibody (**E**) (n = 8). Shown right are representative photographs. *p < 0.05; p < 0.01; one sample* t*-test (A); two-tailed paired *t*-test (B, C, E) and one-tailed paired *t*-test (D).

The inflammation-preventing property of Romidepsin observed in both human and mouse arterial EC motivated us to investigate the potential effect of HDAC1/2 inhibition on the development of diet-induced atherosclerosis in *Apoe*^-/-^ mice. Similar to the observations in the cultured cells, VCAM-1 and GATA6 expression were inhibited in the thoracic abdominal artery from mice intraperitoneally injected with Romidepsin (Figure 6A). The Romidepsin treatment caused no significant change in body weight, serum lipid or glucose levels (Figure S4A-B). However, Romidepsin treatment significantly attenuated lesion formation in the whole *en face* aortas by 46% (Figure 6B), concomitant with reduced lesion size in the aortic sinus (Figure 6C) and aortic arch (Figure 6D) by 18% and 35%, respectively. Furthermore, the lipid deposition as indicated by oil-red O staining was also decreased (data not shown). Consistent with the anti-adhesive effect observed *in vitro*, Romidepsin treatment decreased the content of macrophages in the plaque by 54% (Figure 6E). These results provide* in vivo* evidence confirming a role for HDAC1/2 in mediating VCAM-1-dependent inflammation, which promotes the development of atherosclerosis.

## Discussion

The role of HDACs in mediating diverse cellular processes has been extensively examined 31 and their global pharmacological inhibition using SAHA has shown potent anti-inflammatory activity in preclinical studies of cardiovascular diseases. However, neither the specific HDACs contributing to atherosclerosis nor the epigenetic mechanisms through which the inhibitors modulate inflammatory function have been revealed. Here we report that Romidepsin, through specific inhibition of HDAC1/2, attenuated TNFα-induced VCAM-1 expression and monocytic adhesion to arterial EC through regulation of STAT3 acetylation and *GATA6* methylation. Consistent with the established role of VCAM-1 in promoting monocyte adhesion and atherogenesis 2, Romidepsin reduced the extent of atherosclerotic lesion and plaque macrophage infiltration in hypercholesterolemic *Apoe^-/-^* mice.

Aberrant epigenetic modulation of gene expression programs has consistently been observed in tissues and cells from patients with atherosclerotic heart diseases, suggesting that atherosclerosis has an epigenetic component and highlighting the therapeutic potential in the development of epigenetic targeting drugs for treatment of cardiovascular disease 3, 4. More recently, a parallel study reported that multiple HDAC isozymes including HDAC1/2 were upregulated in advanced human atherosclerotic lesions, and the nonselective pan-HDAC inhibitor SAHA significantly reduced the progression of atherosclerotic lesions in the aorta of high-fat and cholesterol-rich diet-fed *Apoe*^-/-^ mice, through a mechanism targeting Nox expression and inflammatory M1 macrophage polarization 16 or activating endothelial KLF-2 32. Our study provided further evidence in support of the potential for epigenetic intervention of atherosclerosis. However, whether Romidepsin also affects endothelial KLF-2 or macrophage function remains to be investigated. As a more specific HDAC1/2 inhibitor, Romidepsin may hold advantage over SAHA for the intervention of atherosclerosis and other vascular inflammatory disorders.

This study identified HDAC1/2 as the specific targets of Romidepsin in suppressing EC activation and atherosclerosis. HDAC1 or HDAC2 alone was previously shown to be sufficient for normal cardiac development. Only deletion of both *Hdac1* and *Hdac2* resulted in pronounced defects in myocardial growth and morphogenesis in mice 33. In a similar manner, we found that knockdown of HDAC1/2 in tandem but not singly rendered significant inhibition of TNFα-induction of GATA6 and VCAM-1. Our observation that siRNA-mediated knockdown of* HDAC1/2* in HAEC (Figure 1) attenuated VCAM-1 expression in a dose-dependent manner, further implies that a small amount of active HDAC1 or 2 might be sufficient to maintain a low methylation state of *GATA6.* The latter might be a necessary mechanism for EC to ensure normal response to various stimuli.

VCAM-1 and ICAM-1 both belong to the immunoglobulin superfamily and promote EC interaction with leukocytes 1. Their expression is regulated by common as well as specific transcriptional mechanisms. Although HDAC1/2 may affect the acetylation state and thus the activity of NF-κB 14, which is a master transcription factor regulating both VCAM-1 and ICAM-1 expression, both NF-κB p65 phosphorylation and its transcriptional activity were not affected by inhibition of HDAC1/2. This was supported by the observation that ICAM-1 expression remained unchanged.

The unique binding sites for transcription factors IRF-1 and GATA6 residing only on the *VCAM1* promoter accounted for differential regulation of VCAM-1 and ICAM-1 expression by natural compounds, cytokines, circulating lipoproteins, or mTOR inhibition 23-26. Knockdown of either IRF-1 or GATA6 in HAEC significantly inhibited VCAM-1 expression 21, 25. Consistently, we found in this study that hypermethylation-mediated transcriptional silencing of *GATA6* contributed to the suppressive effect of HDAC1/2 inhibition on VCAM-1 but not ICAM-1 expression. This resonates with a previous discovery that pharmacological inhibition of either global HDAC (with trichostatin A) or GATA (with K-11430) attenuated TNFα-induced *VACM1* but not *ICAM1* transcription 34. HDAC3/5/7 were reported to modulate the EC response to different flow patterns through post-transcriptional regulation of GATA6 by microRNA 35. However, a decrease in both steady-state mRNA and hnRNA confirmed that inhibition of HDAC1/2 silenced *GATA6* transcription. Hypermethylation of the *GATA6* promoter was observed in the epigenetic signatures of glioblastoma 36 and gastric cancer 37. Interestingly, our study for the first time demonstrated epigenetic modification of *GATA6* in the regulation of VCAM-1 surface expression by cytokine-activated EC as relates to atherogenesis. In accordance with the previous research reporting that inhibition of GATA6/VCAM-1 contributed to the anti-atherosclerotic effect of RARα/RXRα agonists in *Apoe*^-/-^ mice 38, the current study revealed that a similar mechanism is shared by both human and mouse arterial EC that also accounts for the beneficial effect of Romidepsin on the suppression of atherosclerosis. It is noteworthy that although GATA6 is commonly observed in the differential regulation of VCAM-1 and ICAM-1 23-26 and -259 binding site on *VCAM1* promoter the main sequence used by EC for GATA6 binding in response to TNFα stimulation 28, other GATA members such as GATA4 39 may also participate in this process through interaction with -259 or other GATA-binding site.

This study further characterized STAT3 as a non-histone substrate of HDAC1/2 in the regulation of GATA6-mediated VCAM-1 expression. STAT3 is a physiologically important cytokine-induced transcription factor, which is subject to multiple posttranslational modifications. In addition to phosphorylation of tyrosine or serine residues, activity of STAT3 is modulated by lysine acetylation 40. STAT3 acetylation further promoted phosphorylation and transcriptional activity in hepatocytes 41, 42. Exchanging arginine for Lys685 has been reported to disrupt STAT3 dimerization leading to impairment in DNA-binding and subsequent transactivation activity 12, 43. The inhibitor of acetylation, Resveratrol, was identified as a STAT3 inhibitor 44, further confirming the importance of acetylation in regulating STAT3 function. However, acetylated STAT3 was recently also shown to silence target gene expression via inducing DNA hypermethylation 15, 37, 45, 46. Consistent with previous reports that histone acetyltransferase p300-mediated STAT3 acetylation on Lys685 was reversible by Class I HDAC 12, 40, we found that selective inhibition of HDAC1/2 significantly induced STAT3 acetylation. Mutating STAT3 at Lys685 decreased *GATA6* promoter methylation and partially reversed Romidepsin-inhibited GATA6 and VCAM-1 expression, which confirmed our hypothesis that inhibition of HDAC1/2 increased STAT3 acetylation to enhance *GATA6* promoter methylation.

Interestingly, we found an inverse correlation between STAT3 phosphorylation at Tyr705 and acetylation at Lys685 in both Niclosamide- and Romidepsin-treated HAEC, suggesting a possible causal relationship between these two modifications. However, this was ruled-out as Niclosamide was still capable of inhibiting STAT3 phosphorylation in HAEC where acetylation was blocked by Lys685 mutation. Similarly, a study in hepatocytes showed that mutation at Tyr705 did not affect the acetylation of STAT3 47. Tyrosine phosphorylation of STAT3 was increased by stimulation with cytokines such as TNFα and IL6 48, 49. Our observation that K685R mutation failed to confer full resistance to the inhibitory effect of Niclosamide on GATA6 expression, implies that both decreased phosphorylation and increased acetylation of STAT3 were responsible for the downregulation of GATA6 and VCAM-1 by HDAC1/2 inhibition. While the interrelationship between STAT3 phosphorylation and acetylation warrants further investigation, our data provided sufficient evidence that STAT3 acetylation contributed to promoter methylation-mediated *GATA6* silencing independent of STAT3 phosphorylation.

STAT3, through its NH2-terminal acetylation domain binds to HDAC1 42, and HDAC1 interacts with HDAC2 to form complexes 50. Our observation that inhibition of HDAC1/2 increased STAT3 binding to both HDAC1/2 and the *GATA6* promoter, might be attributed to an accumulation of total and acetylated STAT3 in the nucleus (Figure S3), as also revealed in HDAC1-silenced cells 42. Acetylation of Lys685 in C-terminal transactivation domain of STAT3 was crucial for promoter hypermethylation-mediated silencing of tumor-suppressor genes 15. This study added *GATA6* to the list of downstream genes affected by STAT3 acetylation. As acetylation of STAT3 at residues Lys49 and Lys87 at the N-terminal are necessary for its interaction with HDAC 41, 42, additional studies are required to elucidate whether these and other acetylation sites of STAT3 are involved in the regulation of* GATA6*.

In contrast to the report that STAT3 acetylation contributed to DNMT1 expression in MEF cells 15, neither mutation of STAT3 at Lys685 nor treatment with Romidepsin significantly altered DNMT1 expression in the current study. We revealed that acetyl STAT3 in HDAC1/2-inhibited EC mediated *GATA6* methylation through interaction with DNMT1. DNMT1 was reported to function as a transcriptional repressor, which depended directly on the activity of HDAC1/2 51, 52. The transcriptional repression activity of DNMT1 could be independent of, or in addition to, its capacity to maintain DNA methylation 52. Our study indicated that inhibition of HDAC1/2 silenced *GATA6* through enhancing methylation; however, a methylation-independent mechanism could not be excluded.

In summary, we provide preclinical evidence that HDAC1/2 contribute to EC activation promoting atherosclerosis, and we reveal a novel epigenetic mechanism by which HDAC1/2 inhibition attenuates this inflammatory response. Specifically, modulation of transcription factor STAT3 acetylation and *GATA6* promoter DNA methylation leads to suppression of EC VCAM-1 that supports monocyte recruitment. These findings motivate further studies to test the efficacy of the clinically available HDAC1/2 inhibitor drug Romidepsin in the intervention of atherosclerotic heart disease and other inflammatory diseases of the vasculature.

## Supplementary Material

Supplementary figures.

## Abbreviations

DNMT1: DNA methyltransferase 1
HDAC1/2: histone deacetylases 1 and 2
HAEC: human aortic endothelial cells
hnRNA: heterogeneous nuclear RNA
ICAM-1: intercellular adhesion molecule-1
MSP: methylation-specific PCR
VCAM-1: vascular cell adhesion molecule-1
