# Supplementary material for: HDAC1 and 2 regulate endothelial VCAM-1 expression and atherogenesis by suppressing methylation of the GATA6 promoter
Source: Theranostics. 2021 Mar 20;11(11):5605–19. doi: 10.7150/thno.55878 (PMC8039941; doi:10.7150/thno.55878)
Supplement: Supplementary file 1 — Supplementary figures. [file thnov11p5605s1.pdf]

HDAC1 and 2 regulate endothelial VCAM-1 expression and atherogenesis by suppressing methylation of the *GATA6* promoter

Chengxiu Hu[1,2#], Kai Peng[1,2#], Qianqian Wu[1,2#], Yiying Wang[1,2], Xing Fan[1,2], Dai-Min Zhang[3], Anthony G. Passerini[4], ChongXiu Sun[1,2*]

[1]Key Laboratory of Targeted Intervention of Cardiovascular Disease, Collaborative Innovation Center for Cardiovascular Disease Translational Medicine, Nanjing Medical University, Nanjing, China

[2]Key laboratory of Human Functional Genomics of Jiangsu Province, Nanjing, China

[3]Department of Cardiology, Nanjing First Hospital, Nanjing Medical University, Nanjing, China

[4]Department of Biomedical Engineering, University of California Davis, Davis CA, USA

# contributed equally to this study.

*Correspondence to:
ChongXiu Sun, PhD, Professor
Key Laboratory of Targeted Intervention of Cardiovascular Disease, Collaborative Innovation Center for Cardiovascular Disease Translational Medicine, Nanjing Medical University, Nanjing

101 Longmian Avenue, Jiangning District, Nanjing 211166, P.R. China
Tel.: 86-25-86869429
Fax: 86-25-86869430
E-mail: cxsun@njmu.edu.cn

Figure S1

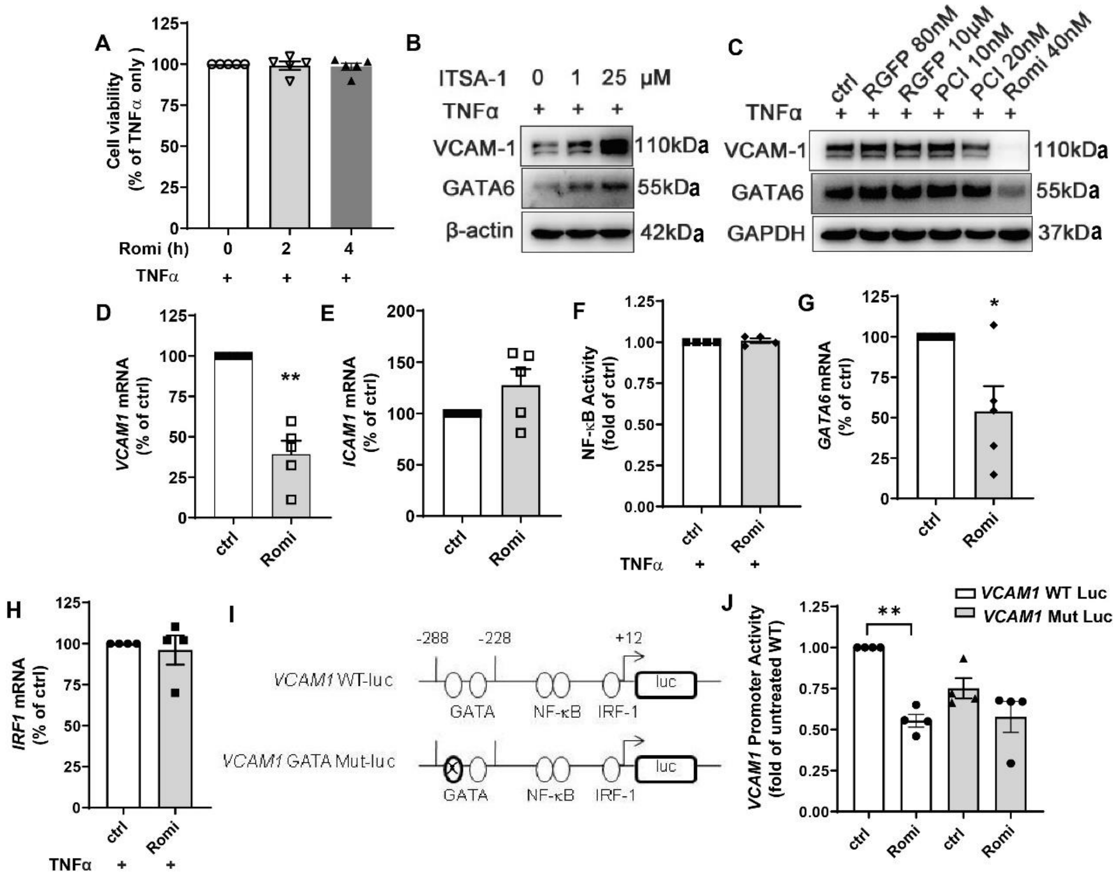

Figure S1. **(A)** HAEC were pretreated with Romi (40 nM) for 1 h, followed by another 2 h or 4 h con-incubation with TNFα (0.1 ng/mL). CCK-8 assay was applied to cell viability (n = 5). **(B)** HAEC were treated with HDAC activator ITSA-1 at indicated concentration, followed by TNFα stimulation prior to Western blot analysis. (**C**) HAEC were pretreated with HDAC3 inhibitor RGFP, HDAC8 inhibitor PCI or HDAC1/2 inhibitor Romi, followed by TNFα stimulation and Western blot analysis. (**D-E**) HAEC were treated with 40 nM Romidepsin (Romi) for 5 h followed by quantitative PCR to measure *VCAM1* (D) or *ICAM1* (E) mRNA (n = 5). (**F**) After treatment with 40 nM Romidepsin for 1 h, HAEC were stimulated with TNFα for 2 h, followed by dual-luciferase assay to detect the transcriptional activity of NF-κB (n = 4). (**G**) HAEC were treated with 40 nM Romidepsin (Romi) for 5 h followed by quantitative PCR to measure *GATA6* mRNA (n = 5). (**H**) After treatment with Romi (40 nM) and TNFα, *IRF1* mRNA was measured (n = 4). (**I**) pGL3 firefly luciferase (luc) constructs of the *VCAM1* promoter (-288/+12) were generated. Mutation (Mut) is depicted by an “X”. (**J**) After

transfection and treated with Romi, the activity of the WT or Mut *VCAM1* promoter construct was measured by the dual-luciferase reporter system (n = 4). *$p < 0.05$; **$p < 0.01$; repeated measures one-way ANOVA followed by Dunnett's test (vs. ctrl, A, J); one sample *t*-test (D, E, F, G, H).

Figure S2

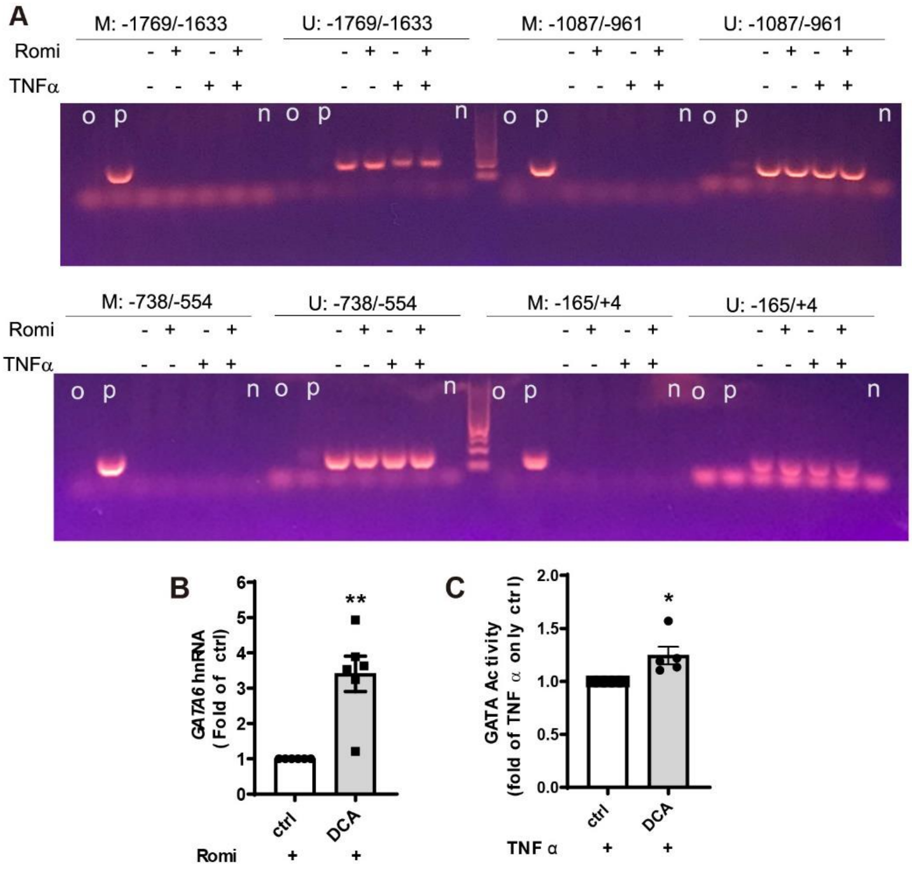

Figure S2. (A) After treatment, *GATA6* promoter methylation in CpG-rich regions -1769/-1633, -1087/-961, -738/-554 and -165/+4 was evaluated by agarose gel electrophoresis detection of MSP products with primers targeting the methylated (M) or unmethylated (U) cytosines. o, original DNA without bisulfite modification; p, positive control using M. SssI-treated peripheral blood leukocyte DNA; n, non-DNA negative control. Shown are representative images of two experiments. **(B)** HAEC were treated with Romidepsin alone or together with DCA (5 μM) followed by hnGATA expression measured as in 2E (n = 6). (**C**) HAEC were pretreated with DCA (5 μM) for 1 h followed by stimulation with TNFα. The transcriptional activity of GATA was examined by dual-luciferase assay (n = 5). *p < 0.05; **p < 0.01; one sample *t*-test (B, C).

Figure S3

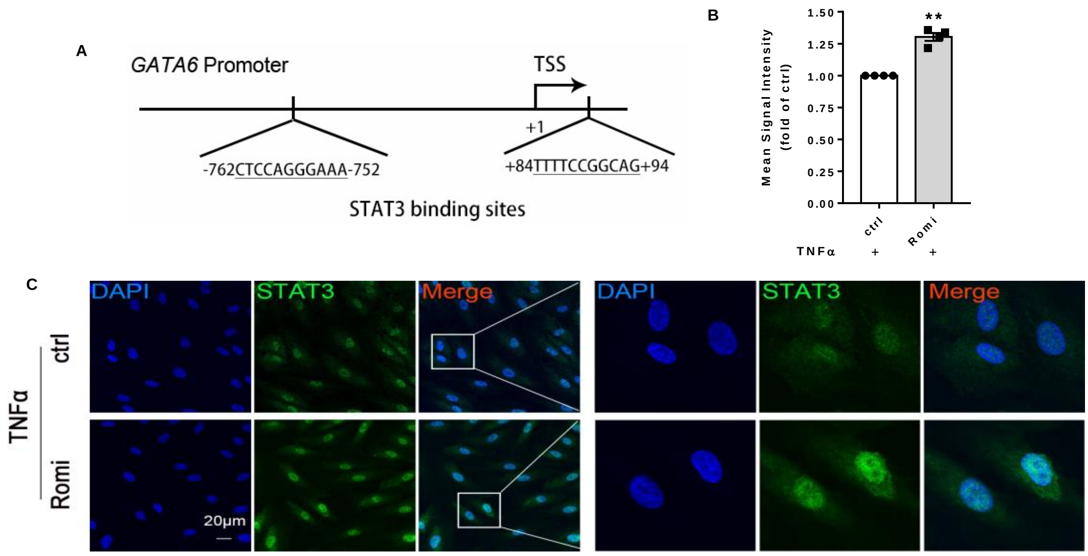

Figure S3. **(A)** Schematic illustrating two predicted STAT3-binding sites on human *GATA6* promoter. (**B-C**) HAEC were grown on coverslips and immunolabeled for STAT3. Images were obtained by a confocal laser scanning microscope (FV1200) and nuclear intensity quantified (B, n = 4). **C**, representative images. **p < 0.01; one sample *t*-test (B).

Figure S4

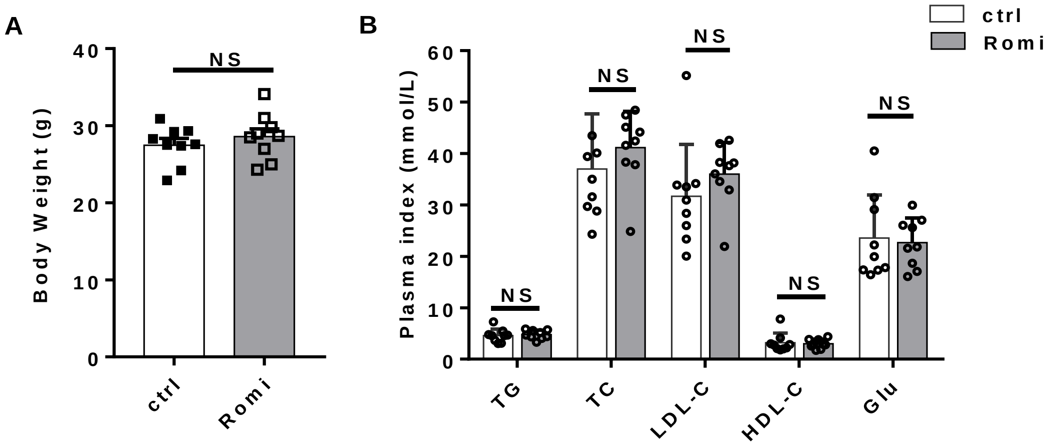

Figure S4. *Apoe*$^{-/-}$ male mice were i.p. injected with Romidepsin (Romi) or PBS (ctrl) and fed with a high-fat diet for 12 weeks (n = 9). Romi treatment caused no significant change in body weight (A), serum lipid or glucose levels (B). TG, triglycerides; TC, total cholesterol; LDL-C, low density lipoprotein cholesterol; HDL-C, high density lipoprotein cholesterol; Glu, glucose. Two-tailed paired *t*-test (A, B).
